# Alignment considerations in degenerative spinal conditions: A narrative review

**DOI:** 10.1016/j.xnsj.2024.100562

**Published:** 2024-10-01

**Authors:** Vincent Challier, Joseph E. Nassar, Jean-Etienne Castelain, Matthieu Campana, Clément Jacquemin, Soufiane Ghailane

**Affiliations:** a Spine Unit, Hôpital privé Francheville Groupe Bordeaux Nord Aquitaine, Hôpital Privé du Dos Francheville, 24000 Périgueux, France; b Brown University Orthopedic Spine Research Unit, Providence RI 02903, United States

**Keywords:** Spinal alignment, Degenerative spine, Degenerative spondylolisthesis, Cervical degenerative myelopathy, Compensatory mechanisms, Aging spine, Spinal deformity

## Abstract

**Background:**

With an aging population, degenerative spinal diseases are contributing significantly to the healthcare's burden. Spinal alignment in the context of adult spinal deformities has become an important domain of research.

**Methods:**

We conducted a narrative review of the latest considerations in spinal alignment within the context of degenerative spinal conditions, discussed current strategies for morphological assessment and finally identified potential areas for future research.

**Results:**

This review reported that degenerative spinal conditions lead to a complex disruption of spinal alignment. It also highlighted the importance of spino-pelvic alignment with specific attention to compensatory mechanisms that occur in response to spinal deformities. Emerging technologies including Artificial Intelligence and epigenetics are showing promises in terms of patient care.

**Conclusions:**

Understanding spinal alignment in degenerative conditions underscores the importance of dynamic and individualized assessments. Future research should integrate emerging technologies along with traditional clinical practices in order to optimize patient outcomes and minimize complications for patients suffering from degenerative spinal diseases.

## Introduction

With the global population rapidly aging, it is reported that by 2050 nearly one-third of the population will be elderly. The United States’ senior care market is valued alone at 1,703.03 billion dollars in 2022 and is projected to reach a staggering 2,882.66 billion dollars by 2030 with a compound growth rate of 6.80% during the forecast period [1,2]. Among the many health issues that come with aging, degenerative spinal conditions, contribute largely to the healthcare burden and constitute a significant challenge to healthcare practitioners [3]. Throughout the past 2 decades, spinal alignment in adult spinal deformities (ASD) has become a critical focus of research driven by the advancements in radiogeometry and based on health-related quality of life (HRQoL) scores [4]. While surgical treatment for ASD has seen remarkable evolution with the advent of classifications and personalized strategies, such care continues to pose substantial risks including mechanical complications which require aggressive revisions [5]. This review aims to investigate the latest considerations in spinal alignment within the context of degenerative spinal conditions, discuss current strategies for morphological assessment, and identify potential areas for future research.

## Degenerative cascade of the aging spine

**Fig. 1:**
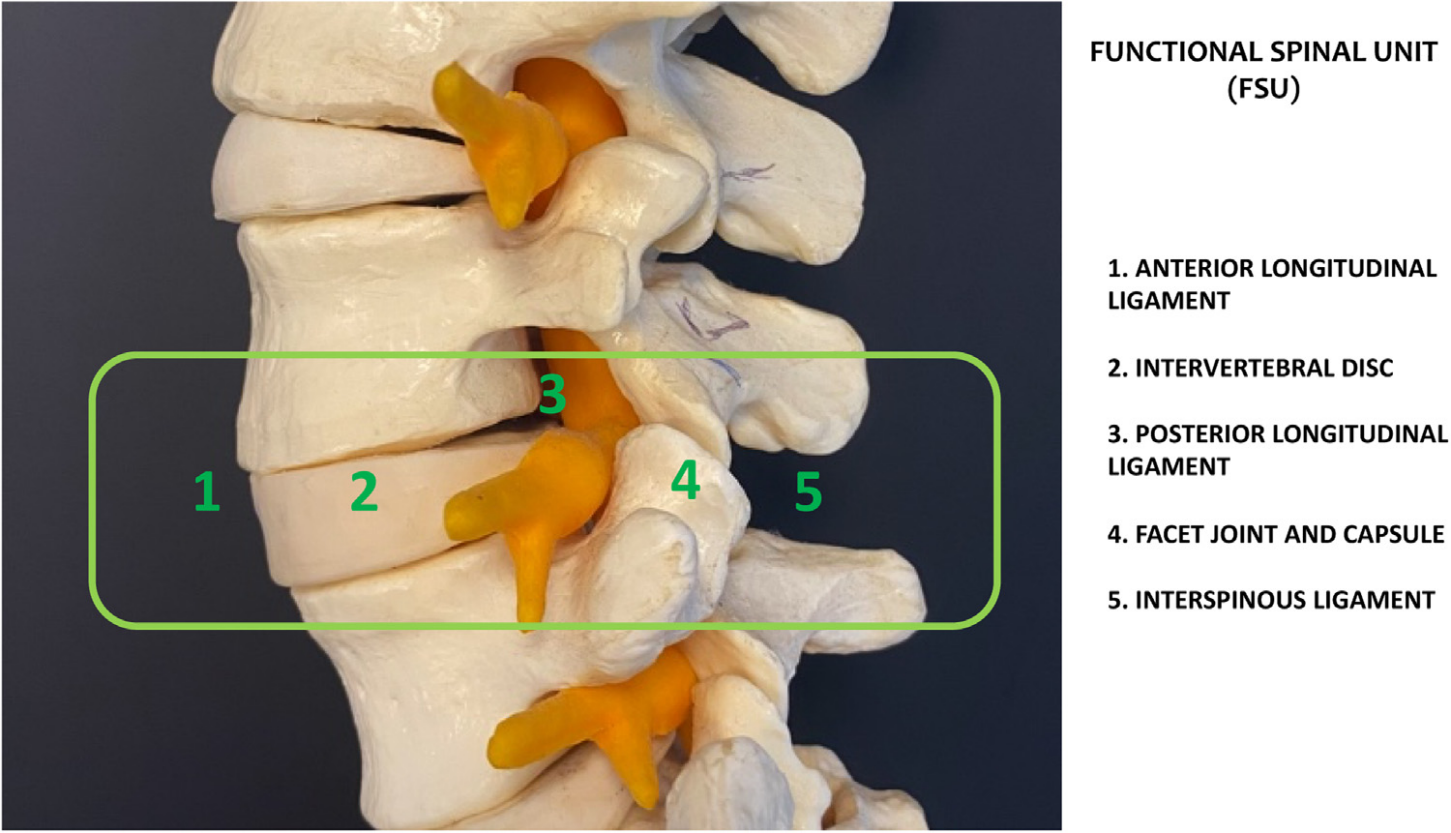
The functional spine unit (FSU).

Aging is closely associated with the progression of degenerative changes within the spine which affects both its structural integrity and functional capabilities. The functional spinal unit (FSU) consisting of bony and soft structures is regulated by neural elements and is positioned between 2 adjacent vertebrae (Fig. 1). This intervertebral unit includes the intervertebral (IVD), ligaments, zygopophyseal capsule and the facet joints (FJ).

### The intervertebral disc (IVD)

The IVD functions as a hydraulic damper and is composed of 2 parts: the outer fibrous nucleus pulposus and the central cartilaginous annulus fibrosus. It is particularly susceptible to degeneration due to its low cell content and fragile avascular tissue, especially in adults [6,7]. Degenerative changes in the IVD referred to as degenerative disc disease (DDD) are considered the driving force behind kyphotic or coronal asymmetric events in the aging spine. These changes begin at the level of the nucleus pulposus and are driven by increased shearing forces that lead to various morphological injuries such as spinal changes, disc-height narrowing, disc bulging, herniations, osteophytes, or endplate changes [[8], [9], [10]]. While these injuries can occur in asymptomatic individuals, they are more prevalent in those who experience pain and disability which typically affect the cervical or lower lumbar levels and often lead to neurological compression [[10], [11], [12]]. Moreover, concomitant DDD in both cervical and lumbar regions is common [13]. Importantly, specific asymmetric alterations in the organization of the cell and the extracellular matrix of the idiopathic scoliotic IVD have been identified to contribute to the progression of the curve [14,15]. The etiopathogenesis of DDD remains unclear, but current research is focusing on mechanical loading, catabolic tissue remodeling due to inflammation and insufficient nutrient supply [16].

### Facet joint (FJ) degeneration

Along with IVD degeneration, alterations in the FJ represent another significant aspect of the degenerative process [17]. As individuals age, FJ osteoarthritis can develop, sometimes leading to pain and contributing to conditions such as degenerative spondylolisthesis (DS) particularly at the level of L4–L5 [[18], [19], [20]]. This condition is also linked to rotatory subluxation, scoliosis, thickening of the ligamentum flavum, and spinal stenosis[21].

### Muscle abnormalities

Muscle atrophy is another component of this degenerative cascade [22]. The European Working Group on Sarcopenia in Older People (EWGSOP) defined sarcopenia as the “progressive and generalized loss of skeletal muscle mass and strength with a risk of adverse outcomes such as physical disability, poor quality of life and death” [23]. This condition typically begins around the age of thirty and can lead to a 30% loss of strength per decade after the age of sixty [7]. In 1983, Hadar et al. described 3 stages of fatty muscle degeneration in lumbar axial CT-scan assessments [24]. Several studies have since shown a correlation between paraspinal muscle atrophy and spinal deformities, particularly when the multifidus and erector spinae in the thoracolumbar region or the cervical extensor musculature are affected [[25], [26], [27], [28], [29], [30], [31]].

### Osteoporosis

Another important factor in spinal degeneration is osteoporosis (OP) which affects nearly 200 million people worldwide, with osteoporotic fractures projected to cost over 25 billion dollars in the U.S. by 2025 [32]. This metabolic disease primarily affects women over 50 by impacting their bone architecture, density, and strength [33,34]. Some researchers have identified oxidative stress as a key factor in the cellular processes involved in OP which is typically diagnosed on dual-energy X-ray absorptiometry (DEXA) with a T-score ≤ -2.5 [35]. However, recent studies have shown strong correlations with Hounsfield Units (HU) in axial L4 CT scans, which may offer a cost-effective substitute for diagnosing OP by reducing time and minimizing expenses [36,37]. One of the first papers discussing global alignment was published by Itoi et al. in 1991 who studied a series of 100 OP patients and found that thoracic kyphosis was the primary deformity that was compensated by a posterior pelvic shift [38]. In a recent review, Najjar et al, confirmed these findings highlighting the loss of lumbar lordosis (LL) along with spinopelvic radiographic parameters as key factors of spinal deformity [39].

Moreover, bones and muscles are deeply interconnected at the biochemical, cellular and tissue levels which led to recognizing osteosarcopenia as an age-related syndrome with strong genetic determinants. This condition is associated with poor health outcomes, an increased risk of institutionalization, falls an fractures [40,41].

### Neurological system decline

The aging of the neurological system is also a significant contributor to the degeneration of the musculoskeletal system with a critical interplay between the central and peripheral neuromuscular systems [[42], [43], [44]]. A typical example of this is camptocormia, characterized by a posture with at least 45° of forward flexion of the trunk while standing, which resolves when lying down in supine position. Common causes of camptocormia include Parkinson's disease, axial myopathy and degenerative spine conditions [45].

In addition to these, neck proprioception also has a vital role in overall balance and the multifactorial decline in cervical range of motion in the elderly increases the risks of falls, which constitute the second leading cause of unintentional traumatic death worldwide [46].

## Spinal alignment as a component of overall balance

**Fig. 2:**
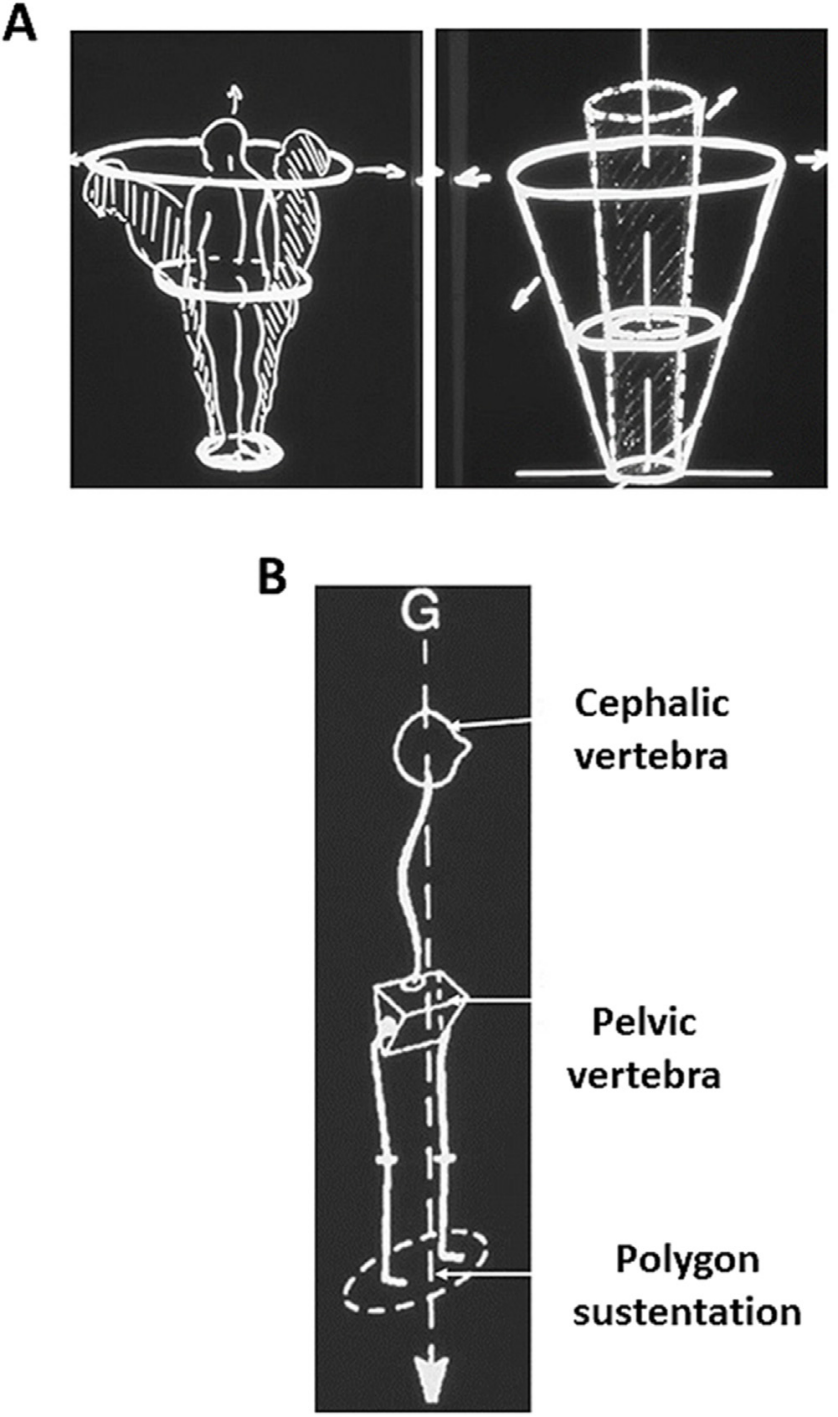
Concept of the cone of economy (A) with the chain of balance (B) [47].

**Fig. 3:**
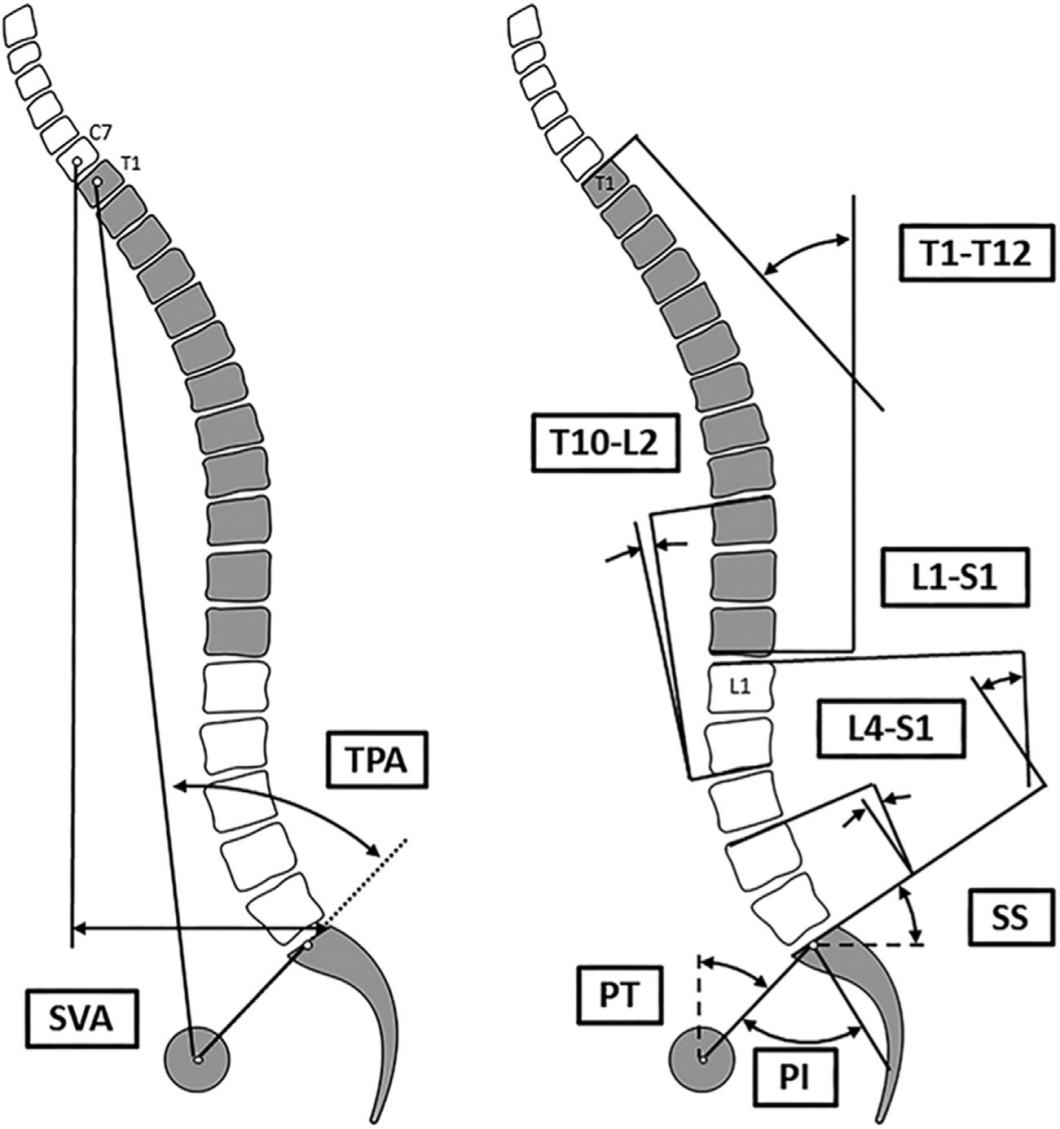
Schematic representation of radiographic parameters used for sagittal alignment assessment [87]. *TPA= T1-pelvic Angle, SVA= sagittal vertical axis, T1-T12= thoracic kyphosis, T10-L2 = thoracolumbar junction, L1-S1 = LL, and L4-S1 = distal LL or lower LL*.

Spinal alignment (SA) plays an important role in the human body's ability to maintain an upright posture and balance. Both are essential for daily activities and to ensure proper overall quality of life. SA refers to the arrangement of the vertebrae and the curvature of the spine. In optimal condition, it allows for the effective distribution of forces and supports the body's weight. The principle of Jean Dubousset's “cone of economy” illustrates the minimal energy expenditure required to maintain an upright posture within a specific spatial area [47] (Fig. 2). Since then, the spinal deformity literature has developed an understanding of several sagittal alignment parameters (Fig. 3). However, SA is only 1 static parameter within the broader concept of overall balance, which is multi-modal and includes neurosensory systems such as vestibular, oculomotor, proprioceptive, and cerebral functions which are all based on 2 fundamental principles: signal and feedback loops as well as neuromuscular effectors that create reactions and motion. Balance which refers to stability within motion, is a complex mechanism that has evolved throughout human history, allowing for bipedalism, horizontal gaze and an erect posture [[48], [49], [50], [51]].

## Three-dimensional spinal alignment

The horizontal plane first described by the French engineer Magny in 1780, is a crucial yet often underestimated component in understanding SA and its impact on both regional and global alignment warrants further investigation [52].

The changes in radiographic SA initially measured by John Cobb (1903–1967) who primarily focused on the coronal plane have been linked to HRQoL in aging individuals with spinal deformities [[53], [54], [55], [56], [57]]. The recent Obeid Coronal Malalignment classification underscores the importance of using bending radiographs to assess for stiffness which is crucial for guiding surgical strategy [[53], [54], [55], [56], [57]].

In contrast, the assessment of static sagittal SA is primarily morphological and involves full-body imaging in a standing position. This assessment considers 3 key modules: spino-pelvic harmony, compensatory mechanisms and posture. These foundational principles of spinal radiogeometry have been progressively described over the last forty years [38,[58], [59], [60]].

Understanding these principles is essential in managing spinal alignment, especially in the context of degenerative spine conditions.

## Basic principles of sagittal alignment and compensatory mechanisms

**Fig. 4:**
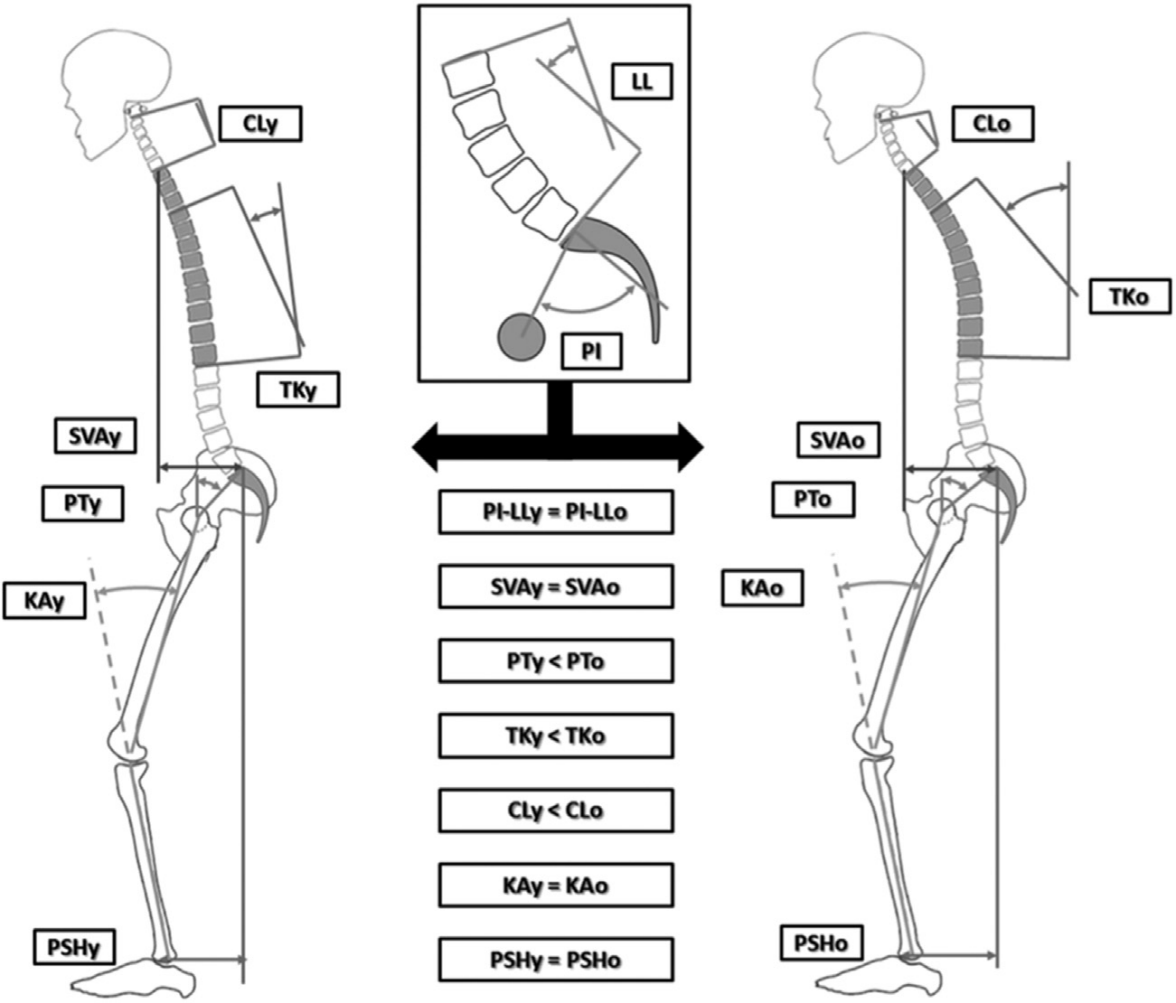
Approximate illustration of 2 kinds of compensatory mechanism recruitment for the same magnitude of spinopelvic mismatch. Left figure represents a young patient who recruited thoracic hypokyphosis as well as pelvic and lower limb mechanisms. Right figure represents older patient with minimal thoracic involvement, therefore more pelvic retroversion and more CL than the younger patient [64]. *CL indicates cervical lordosis; KA, knee flexion angle; LL, lumbar lordosis; o, older; PI, pelvic incidence; P.Sh, pelvic shift; PT, pelvic tilt; SVA, sagittal vertical axis; TK, thoracic kyphosis; y, younger.*

A key aspect of SA is spino-pelvic harmony, which refers to the natural curvature of the spine in relation to the shape of the pelvis [61]. The relationship between Lumbar lordosis (LL), a dynamic module, and pelvic incidence (PI), a relatively fixed parameter, is particularly important in maintaining this harmony [62,63]. Degenerative conditions that cause a loss of LL can disrupt this balance triggering a forward shift of the trunk which is usually compensated by thoracic hypokyphosis, increased pelvic tilt (PT), and knee flexion [64,65] (Fig. 4). The sagittal shape of the pelvis, as measured by the PI, predicts the reserve for pelvic retroversion. In fact, individuals with a high PI can increase their PT and the opposite is true for those with a low PI [66,67].

In some cases, the disruption of spino-pelvic harmony originates in the thoracic region, with conditions such as hyperkyphosis due to traumatic fracture, spondyloarthropathy, or Scheuermann's disease. These are often compensated by lumbar hyperlordosis and pelvi-femoral adaptations, as mentioned earlier. The behavior of compensatory mechanisms, particularly pelvic retroversion after surgery, remains unclear and its multifactorial aspects are currently under scientific investigation to explain the high variability among patients [64,68,69].

**Fig. 5:**
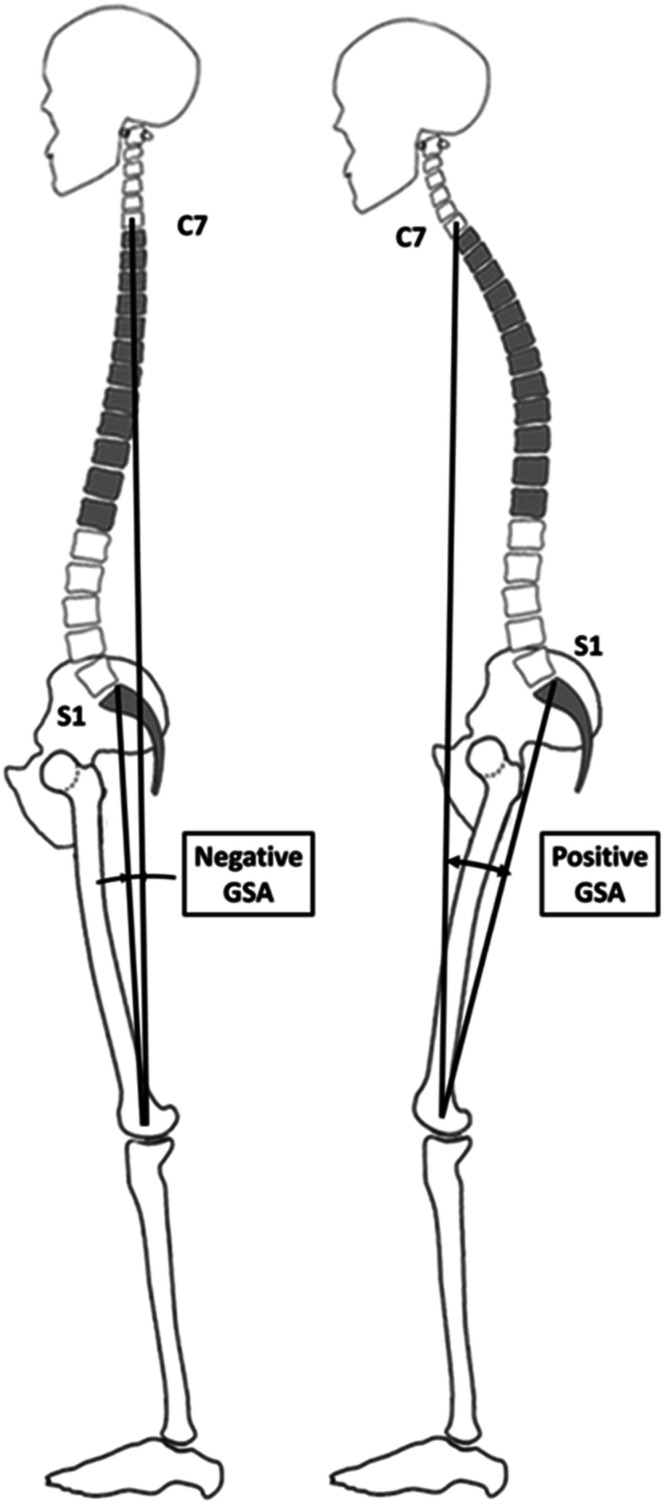
Illustrations showing the GSA: negative value (left) and positive value (right) [73]. *GSA, Global Sagittal Axis.*

To maintain a horizontal gaze, cervical lordosis increases as the final compensatory mechanism when others are exhausted [[70], [71], [72]]. Global alignment assessed by Sagittal Vertical Axis and Global Sagittal Axis, is the result of harmony and compensation [57,73] (Fig. 3, Fig. 5). However, asymptomatic elderly individuals often experience spinal malalignment changes. In fact, Bassani et al. found a 27% rate of scoliosis in a series of 160 elderly volunteers free of symptom [[74], [75], [76], [77]].

## Classifications in sagittal alignment

### Numerical hypothesis and spino-pelvic shape principle

**Fig. 6:**
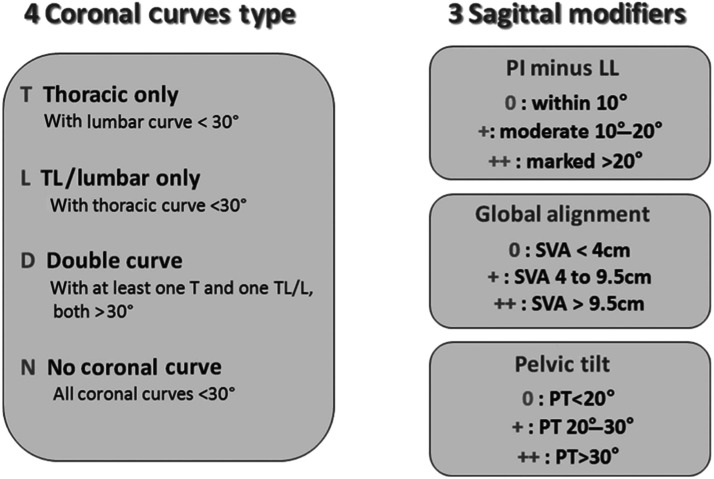
The SRS-Schwab Classification includes 4 coronal curves type and 3 sagittal modifiers [130]. *PI, pelvic incidence; LL, lordosis between L1 and S1; PT, pelvic tilt; SVA, sagittal vertical axis; TL, thoracolumbar.*

**Fig. 7:**
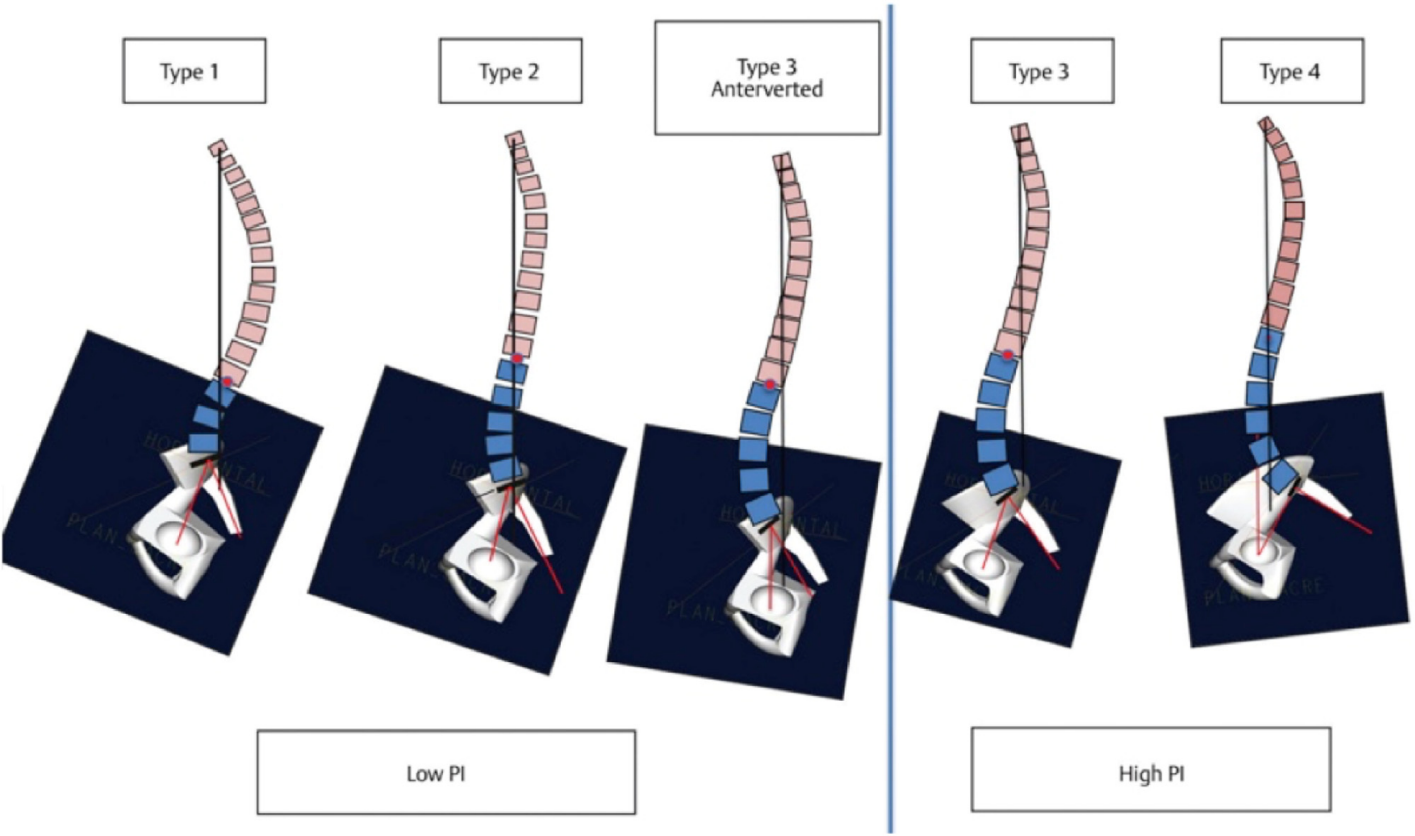
Roussouly Classification[131]. *PI: Pelvic Incidence, low PI<50°, high PI>50°*.

Over the past twenty years, 2 main philosophies have emerged in the context of sagittal alignment. The first is the Numerical Hypothesis which is based on correlations between radiogeometry and HRQoL scores and is supported by publications from the International Spine Study Group (ISSG) and built upon the SRS-Schwab classification [61,[78], [79], [80]] (Fig. 6). The second philosophy is the Spino-Pelvic Shape Principle, which defines 5 types of contours each associated with different patterns of degenerative changes and specific correction strategies as outlined in the Roussouly classification [[81], [82], [83], [84], [85]] (Fig. 7).

One should understand both philosophies to fully grasp the concept of sagittal alignment and its implications in therapeutic strategies. Moreover, mastering both approaches also foster a harmonious communication in meetings while also helping build meaningful scientific connections between different schools of thought [86].

### Advances in segmental sagittal alignment

The recent development of segmental sagittal alignment is bridging the gap between these theories. This approach highlights the segmentation of LL into cranial and caudal areas, defines the thoracolumbar inflexion point and ideal apex, and identifies the inner components of local deformity drivers and compensatory mechanisms [85,[87], [88], [89]].

This understanding is essential for improving surgical planning and help mitigate avoidable mechanical complications such as adjacent segment disease, rod breakage, and proximal junctional kyphosis [85,[87], [88], [89]].

### Cervical sagittal alignment

The cervical region can be apprehended as a neurosensory rod that is highly mobile, guided by horizontal gaze during walking and deeply interconnected with the entire skeleton [90]. Therefore, during the assessment of cervical alignment, the surgeon should highly consider a full-body image [91].

**Fig. 8:**
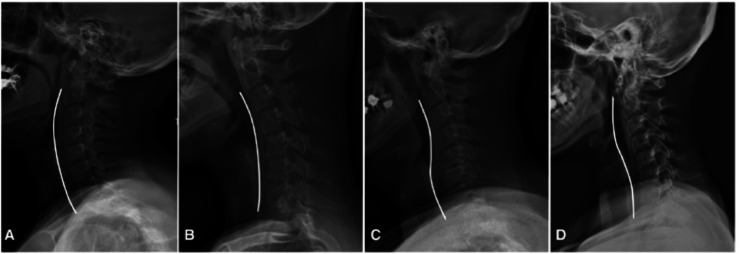
Morphologic variations of cervical alignment patterns: global lordosis (A), global kyphosis (B), sigmoid shape with proximal kyphosis and distal lordosis (C), sigmoid shape with proximal lordosis and distal kyphosis (D) [71].

In 2022, the French Spine Society investigated the prevalence of cervical alignment variations in a population of 2,599 individuals all of whom had no or minor degenerative changes and no prior history of cervical surgery. It identified 4 different patterns of cervical alignment: 50.9% experienced global lordosis, 1.3% had global kyphosis, 34.4% exhibited a sigmoid shape with proximal junctional kyphosis and the remaining 13.4% exhibited a sigmoid shape with distal kyphosis (Fig. 8). These findings highlight the significant variability in cervical alignment [71].

The cervical caudal arch (C2–C7) increased with age, while the cranial arch (C0–C2) remained stable, revealing the cervical region's adaptation to the progressive forward shift of the trunk. This adaptation is also associated with an increase in the slope of the cervico-thoracic junction to preserve horizontal gaze [71].

Ethnicity seems to impact variations in cervical alignment worldwide. In fact, a study conducted by Yukawa et al. in 2012 a 13.4% prevalence of kyphotic alignment in a Japanese population with rates reaching up to 33.3% in 20-year-old females [92]. Moreover, radiographic assessment of the cervical spine in the sagittal view, using maximum flexion and extension, a so-called “dynamic evaluation,” defines a range of motion (ROM) that decreases with increasing age [92,93]. A comprehensive classification of cervical deformity has been proposed by Scheer, in relation to HRQoL [94].

### Spinal alignment and specific degenerative conditions

SA is a fragile homeostasis that can be gradually disrupted by early degenerative conditions. It's distribution also serves as a predictor for the development of specific disorders. In 2007, Barrey et al. compared the sagittal spino-pelvic alignment of 85 patients undergoing surgery for degenerative lumbar disease to that of 154 asymptomatic volunteers [95]. They found that PI was significantly higher in cases of degenerative spondylolisthesis and lower in degenerative disc disease and herniated disc among patients under the age of 45, highlighting the importance of this fundamental parameter in spinal organization as well as its role as a predictor of degeneration [95].

#### Contact forces theory and spino-pelvic shape

The Contact Forces Theory, put forward by Roussouly et al. describes the mechanical loading in the FSU and its interaction with FJ and IVD during flexion-extension movements. This theory underscores the relationship between spino-pelvic shape and degeneration patterns [67,96,97]. Local stress increases in the posterior arch (i.e., FJ) during extension as segmental lordosis increases and a similar effect occurs in the anterior column (i.e., IVD) during flexion in a flat-back shape [98]. In the hypercurved type (Roussouly type 4), FJ degeneration and hyperloading together lead to the development of instability and arthritis resulting in degenerative spondylolisthesis at the L4-L5 level as a typical example. In the flat-back shape (Roussouly type 2), degeneration mainly affects the IVD, leading to DDD [98]. In thoracolumbar kyphotic individuals with a low apex (Roussouly type 1), local stresses usually occur in the upper arch of the lumbar segment (i.e., L1–L2 and L2–L3), and FJ degeneration is common in L4-L5 and L5-S1, with an increased risk of L5-S1 isthmic lysis due to the “nutcracker” effect [98]. This theory has been partially supported by biomechanical in vitro testing and finite element analysis, but still requires confirmation by conducting large population longitudinal studies [99,100].

#### Neurological impairment and sagittal posture

Neurological impairment caused by central stenosis is also known to result in sagittal forward posture as a compensatory mechanism in order to open the spinal canal. This was demonstrated by the famous Van Gelderen cycle and was confirmed by recent studies [[101], [102], [103], [104]].

#### Degenerative lumbar spondylolisthesis

Degenerative lumbar spondylolisthesis lies at the crossroads of ASD and degeneration. Common features include increased PI, regional deformity with loss of caudal LL, and compensation with an increased cephalad LL and PT. Classifications have been proposed regarding local, regional and global malalignment to guide appropriate surgical strategies [[105], [106], [107], [108]].

#### Degenerative cervical myelopathy

Cervical degenerative conditions are influenced by local, regional and global malalignment. Extensive research has been conducted on cervical degenerative myelopathy (CDM) and its relationship to alignment [[109], [110], [111], [112], [113], [114]]. As the leading cause of spinal cord impairment in the elderly worldwide, CDM is characterized by cord compression due to circumferential canal narrowing, along with biochemical degenerative changes in the IVD, hypertrophy of the ligamentum flavum and laxity of FJ [109,110,112]. Both static and dynamic stenosis are involved and correlations between spinal cord volume, hyperintensity on MRI, cervical alignment, and HRQoL scores suggest that positive alignment, decreased ROM, and local kyphotic changes are aggravating factors [109,110,112].

## Emerging technologies in spinal alignment and degenerative conditions

As the world of science is going through a paradigm shift with the emergence of artificial intelligence (AI), the study of SA in the context of degenerative conditions of the FSU is still in its infancy. A notable work published by Sparrey et al. in 2014 reviewed the different parameters of lordosis through evolutionary medicine, mechanobiology, environmental factors and genetics highlighting the importance of dynamic morphological assessment of the skeleton in different postures and movements for future research [115]. Several methods have been proposed recently including gait analysis, active tests, and spinopelvic kinematics systems in the context of ASD patients, showing promising results [[116], [117], [118], [119], [120], [121], [122]]. Moreover, epigenetics has also made innovative progress in frailty assessment and complication prediction [123,124].

Finally, AI is still in its early stages with proof-of-concept studies emerging in areas such as adult spinal deformity imaging, patient triage and surgical strategy [[125], [126], [127], [128], [129]]. Applying AI to large, multi-continental datasets that combine demographic, clinical, morphological and socio-economic analyses will probably provide the granularity needed to enable practitioners to offer multimodal assessments and personalized care.

## Conclusion

Understanding SA in the context of degenerative spinal conditions highlights the complex interchange between anatomical, biomechanical and neurosensory systems that are necessary for older adults to maintain appropriate balance and posture. As the number of elderly people rises worldwide it is becoming more important to understand the factors that contribute to the development of ASD. This review shows the importance of both dynamic and individualized assessments when managing degenerative spinal conditions while taking into account the compensatory mechanisms involved. Moreover, emerging technologies particularly in the fields of AI and epigenetics are showing promising paths for improving both treatment strategies and diagnostic precision. To optimize patient outcomes, reduce complications and eventually raise the quality of life for patients suffering from degenerative spinal disorders, future research should focus on integrating these innovative approaches with their traditional clinical practices.

## Declaration of competing interest

The authors declare that they have no known competing financial interests or personal relationships that could have appeared to influence the work reported in this paper.
